# Genomic distance under gene substitutions

**DOI:** 10.1186/1471-2105-12-S9-S8

**Published:** 2011-10-05

**Authors:** Marília D V Braga, Raphael Machado, Leonardo C Ribeiro, Jens Stoye

**Affiliations:** 1 Instituto Nacional de Metrologia, Qualidade e Tecnologia, Duque de Caxias, 25250-020, Brazil; 2 AG Genominformatik, Technische Fakultät, Universität Bielefeld, Bielefeld, 33594, Germany

## Abstract

**Background:**

The distance between two genomes is often computed by comparing only the common markers between them. Some approaches are also able to deal with non-common markers, allowing the insertion or the deletion of such markers. In these models, a deletion and a subsequent insertion that occur at the same position of the genome count for two sorting steps.

**Results:**

Here we propose a new model that sorts non-common markers with substitutions, which are more powerful operations that comprehend insertions and deletions. A deletion and an insertion that occur at the same position of the genome can be modeled as a substitution, counting for a single sorting step.

**Conclusions:**

Comparing genomes with unequal content, but without duplicated markers, we give a linear time algorithm to compute the genomic distance considering substitutions and double-cut-and-join (DCJ) operations. This model provides a parsimonious genomic distance to handle genomes free of duplicated markers, that is in practice a lower bound to the real genomic distances. The method could also be used to refine orthology assignments, since in some cases a substitution could actually correspond to an unannotated orthology.

## Background

The genomic distance is often computed taking into consideration only the common markers, that occur in both genomes [1-3]. Approaches to deal with unique markers (that occur in only one genome) also exist, but usually allowing only insertions or deletions of these markers. Insertions and deletions can be shortly called *indels*. In [4], the operations allowed are inversions and indels, while the models given in [5] and [6] consider indels and the double cut and join (DCJ) operation [7], that is able to represent most large scale mutation events in genomes, such as inversions, translocations, fusions and fissions. The mentioned approaches assign the same weight to all rearrangement operations, including indels, regardless of the size of the affected regions and the particular types of the operations. A drawback in these models is that, if a deletion and a subsequent insertion occur at the same position of the genome, the cost is the same as a deletion and an insertion in different positions.

**Figure 1:**
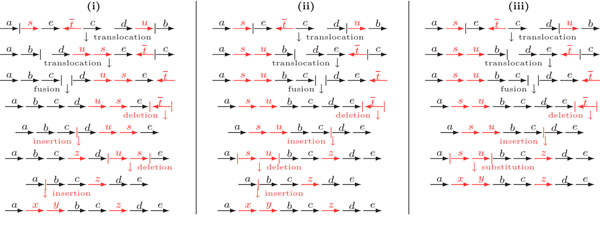
(i) An optimal sorting scenario with DCJ operations and indels. (ii) An optimal sorting scenario with DCJ operations and indels in which the last two operations occur in the same position of the genome, between markers *a* and *b*. (iii) A more parsimonious alternative to the deletion of consecutive markers *s* and *u* and the insertion of consecutive markers *x* and *y* would be the substitution of *s* and *u* by *x* and *y*.

In the present work we propose a more parsimonious model in which, instead of deleting or inserting, we allow the substitution of unique markers between two genomes, as illustrated in Figure 1. We do not suggest that a substitution occurs in a precise moment in evolution, but instead it represents a region that underwent continuous mutations (duplications, losses and gene mutations), so that a group of genes is transformed into a different group of genes (either of which may also be empty, allowing a substitution to represent an insertion or a deletion). Other studies also represent continuous mutations as a rearrangement event [8,9]. By minimizing substitutions we are able to establish a relation between indels that could have occurred in the same position of the compared genomes, identifying genomic regions that could be subject to these continuous mutations. Observe that we suggest that such regions have a common evolutionary origin. We develop a method to count the minimum number of substitutions that could have occurred, by assigning the same weight to substitutions and to the other operations, similarly to the approaches that handle indels.

We analyze genomes with unequal content, but without duplicated markers and extend the results given in [6] to develop a linear time algorithm that exactly computes the genomic distance with substitutions and DCJ operations. The objective of this model is to provide a parsimonious genomic distance to handle genomes free of duplicated markers, that in practice is a lower bound to the real genomic distances. In the present work, we do not study algorithms to generate parsimonious sorting scenarios. Nevertheless, in the analysis of the evolution of human chromosomes X and Y, we manually obtain a parsimonious evolutionary scenario under our model, that is coherent with the results given in [10].

In the remainder of this section we introduce some concepts given in [1] and [6] and define the operation that substitutes markers in a genome - these are the basis of the method that we will present here.

### Preliminaries

In the present study duplicated markers are not allowed. Given two genomes *A* and *B*, possibly with unequal content, we denote by  the “reduced” genome [4], that is the set of markers that occur once in *A* and once in *B*. Moreover, the set  contains the markers that occur only in *A* and the set  contains the markers that occur only in *B*. The markers in sets  and  are also called *unique markers*. Observe that the sets ,  and  are disjoint.

**Figure 2:**
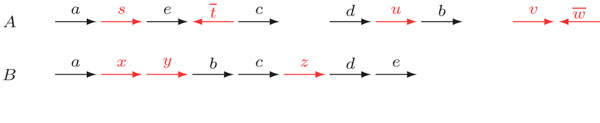
For genomes A, composed of three linear chromosomes, and *B*, composed of one single chromosome, we have ,  and .

A genome is possibly composed of linear and circular chromosomes. Each marker *g* in a genome is a DNA fragment and is represented by the symbol *g*, if it is read in direct orientation, or by the symbol , if it is read in reverse orientation. An example of a pair of genomes is given in Figure 2.

In the following we adopt definitions which we have given in [6] (some of them are generalizations of concepts introduced by Bergeron *et al*. [1]).

### -adjacencies

Each one of the two ends of a linear chromosome is called a *telomere* and is represented by the symbol ○. For each marker , denote its two extremities by *g^t^* (tail) and *g^h^* (head). A *-adjacency* in genome *A* (respectively in genome *B*) is in general a linear string *v* = *γ*_1_*ℓγ*_2_, such that *γ*_1_ and *γ*_2_ are telomeres or extremities of markers of  and *ℓ*, the string composed of the markers that are between *γ*_1_ and *γ*_2_ in *A* (respectively in *B*), contains no marker that also belongs to . The string *ℓ* is said to be the *label* of *v*, and the extremities *γ*_1_ and *γ*_2_ are said to be *-adjacent*. If *ℓ* is a non-empty string, *v* is said to be *labeled*, otherwise *v* is said to be *clean*.

A -adjacency *γ*_1_*ℓγ*_2_ can also be represented by . Furthermore, ◦*ℓ*◦ represents a linear chromosome composed only of markers that are not in . In the same way, a -adjacency given by a label *ℓ* corresponds to a whole circular chromosome composed only of markers that are not in . This is the only case of a -adjacency in which we have a circular instead of a linear string.

Two genomes *A* and *B* can then be represented by the sets  and , containing their -adjacencies. For the two genomes in Figure 2, we have ,  and .

### The DCJ operation

A *cut* performed on a genome *A* separates two adjacent markers of *A*. A cut affects a -adjacency *v* of  as follows: if *v* is linear, the cut is done between two symbols of *v*, creating two open ends in two separate linear strings; if *v* is circular, the cut creates two open ends in one linear string. A *double-cut and join* or DCJ applied on a genome *A* is the operation that generally performs two cuts in , creating four open ends, and joins these open ends in a different way. A DCJ operation can correspond to several rearrangement events, such as an inversion, a translocation, a fusion, or a fission [7].

We represent by ({γ_1_ℓ_1_|ℓ_4_γ_4_ , γ_3_ ℓ_3_ | ℓ_2_γ_2_ } → {γ_1_ℓ_1_| ℓ_2_ γ_2_, γ_3_ ℓ_3_|ℓ_4_ γ_4_ }) a DCJ applied on γ_1_ℓ_1_ℓ_4_γ_4_ and γ_3_ℓ_3_ℓ_2_γ_2_ , that creates γ_1_ℓ_1_ℓ_2_γ_2_ and γ_3_ℓ_3_ℓ_4_γ_4_. Observe that one or more extremities among γ_1_, γ_2_, γ_3_ and γ_4_ can be equal to ○ (a telomere), as well as one or more labels among ℓ_1_, ℓ_2_, ℓ_3_ and ℓ_4_ can be equal to ε (the empty string). Particular cases include circular adjacencies and are described in [6].

### Adjacency graph and the DCJ distance

The *adjacency graph AG*(*A*, *B*) [*1*] is the bipartite graph that has a vertex for each -adjacency in  and a vertex for each -adjacency in . Then, for each , we have one edge connecting the vertex in  and the vertex in  that contain *g^h^* and one edge connecting the vertex in  and the vertex in  that contain *g^t^*.

**Figure 3:**
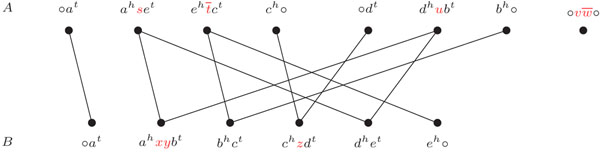
For genomes *A* and *B*, the adjacency graph contains one cycle, two *AA*-paths (one is a linear singleton) and two *AB*-paths.

The connected components of the graph *AG*(*A*, *B*) are cycles and paths that alternate vertices in  and . A path that has one endpoint in  and the other in  is called an *AB-path*. In the same way, both endpoints of an *AA-path* are in , as well as both endpoints of a *BB-path* are in . Furthermore, *AG*(*A*, *B*) can have two extra types of components: each -adjacency that corresponds to a linear (respect. circular) chromosome is a *linear* (respect. *circular*) *singleton*. Linear singletons are particular cases of AA-paths and BB-paths. An example of an adjacency graph is given in Figure 3.

The number of AB-paths in *AG*(*A*, *B*) is always even and a DCJ operation can be of three types [1,6]: *optimal* when it either increases the number of cycles by one, or the number of AB-paths by two; *neutral* when it does not affect the number of cycles and *AB*-paths; or *counter-optimal* when it either decreases the number of cycles by one, or the number of *AB*-paths by two.

Singletons, *AB*-paths composed of one single edge, and cycles composed of two edges are said to be *DCJ-sorted*. Longer paths and cycles are said to be *DCJ-unsorted*. The procedure of using DCJ operations to turn *AG*(*A*, *B*) into DCJ-sorted components is called *DCJ-sorting* of *A* into *B*. The *DCJ distance* of *A* and *B*, denoted by *d_DCJ_*(*A*, *B*), corresponds to the minimum number of steps required to do a DCJ-sorting of *A* into *B* and can be easily obtained:

**Theorem 1** ( [1]**)***Given two genomes A and B without duplicated markers*, *we have*, *where**is the set of common markers between A and B*, *and c and b are the number of cycles and of AB-paths in AG*(*A*, *B*).

### Runs of unique markers

**Figure 4:**
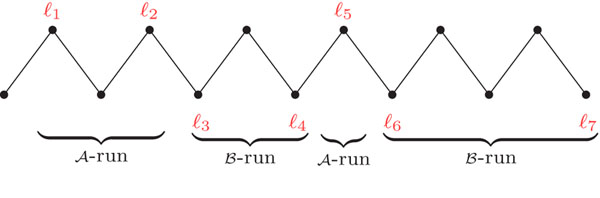
A *BB*-path with 4 runs. Only the labels of the -adjacencies are represented.

Given a component *C* of *AG* (*A*, *B*), we can obtain a string *ℓ*(*C*) by the concatenation of the labels of the -adjacencies of *C* in the order in which they appear. Cycles, *AA*-paths and *BB*-paths can be read in any direction, but *AB*-paths should always be read from *A* to B. If *C* is a cycle and has labels in both genomes *A* and *B*, we should start to read in a labeled -adjacency *v* of *A*, such that the first labeled vertex before *v* is a -adjacency in *B*; otherwise *C* has labels in at most one genome and we can start anywhere. Each maximal substring of *ℓ*(*C*) composed only of markers in  (respectively in  is called an *-run* (respectively a *-run*). Each -run or -run can be simply called *run*[6]. A component composed only of clean -adjacencies has no run and is said to be *clean*, otherwise the component is *labeled*. We denote by Λ(*C*) the number of runs in a component *C*. A path can have any number of runs, while a cycle has zero, one, or an even number of runs. Figure 4 shows a *BB*-path with 4 runs.

### Substitutions

The unique markers in  and  are represented in *AG* (*A*, *B*) as labels and singletons and, in order to sort *A* into *B*, they also have to be considered. Here we propose a model in which only the following operation can be applied to unique markers. A *substitution* is an operation that affects the label of one single -adjacency, by substituting contiguous markers in this label.

Consider the labels *ℓ*_1_ and *ℓ*_2_, where |*ℓ*_1_*|* = *m* and |*ℓ*_2_*|* = *n*. The substitution of *ℓ*_1_ by *ℓ*_2_ in a -adjacency is represented by (*γ*_1_*ℓ*_3_|*ℓ*_1_|*ℓ*_4_*γ*_2_ → *γ*_1_*ℓ*_3_|*ℓ*_2_|*ℓ*_4_*γ*_2_) (for better reading in our notation we omit the curly set brackets for singleton sets). One or both extremities among *γ*_1_ and *γ*_2_ can be equal to ○ (a telomere), as well as one or both labels among *ℓ*_3_ and *ℓ*_4_ can be equal to *ε* (the empty string). The substitution of *ℓ*_1_ by *ℓ_2_* in a circular singleton is represented by (|*ℓ*_1_|*ℓ*_3_| → |*ℓ*_2_|*ℓ*_3_|). Observe that at most one chromosome can be entirely substituted at once (but we do not allow the substitution of a linear by a circular chromosome and *vice-versa*). Moreover, if *m* = 0, we have an *insertion* of *n* contiguous markers. On the other hand, if *n* = 0, we have a *deletion* of *m* contiguous markers. Thus, insertions and deletions, also called *indels*, are special cases of substitutions.

The *DCJ-substitution distance* of *A* and *B*, denoted by , is the minimum number of DCJs and substitutions required to transform *A* into *B*. Since substitutions include indels,  is upper bounded by the *DCJ-indel distance*, the minimum number of DCJ and indel operations required to transform *A* into *B*, that can be computed in linear time [6]. In the present work we give an approach to exactly compute  also in linear time.

## Results and discussion

The main result of the present study is an exact formula to compute the DCJ-substitution distance in linear time. We achieve this formula by developing the substitution-potential of two genomes, a property that allows us to obtain a good upper bound to the genomic distance with DCJ operations and substitutions. Then we show how some special DCJ operations reduce the overall number of substitutions and obtain the exact formula. Although the objective of this model is to provide a parsimonious genomic distance, that in practice is a lower bound to real distances, we run some experiments on data from human X and Y chromosomes and obtained a parsimonious sorting scenario that is coherent with the results available in the literature. We also observe that the DCJ-substitution method could be used to refine orthology assignments.

### The substitution-potential

Observe that a -adjacency with a non-empty label *ℓ* can be cut in at least two different positions, either before or after *ℓ*. Since the position of the cut does not change the effect of the DCJ on *d*_DCJ_(*A*, *B*), we can choose to cut at positions that allow the concatenation of the labels of the original -adjacencies. As a consequence, a set of labels of one genome can be *accumulated* with DCJ operations. In particular, when we apply optimal DCJs on only one component of the adjacency graph, we can accumulate an entire run in a single -adjacency:

**Proposition 1** ( [6]**)***A run can be entirely accumulated in the label of one single**-adjacency with optimal DCJ operations*.

Given a DCJ operation *ρ*, let Λ_0_ and Λ_1_ be, respectively, the number of runs in *AG* (*A*, *B*) before and after *ρ*. We define ∆Λ(*ρ*) = Λ _1_*–* Λ _0_.

**Proposition 2** ( [6]**)***Given any DCJ operation ρ*, *we have* ∆Λ(*ρ*) ≥ – 2.

In order to obtain the exact formula for the DCJ-substitution distance, we will first analyze the components of the adjacency graph separately. Given two genomes *A* and *B* and a component *C* ∈ *AG* (*A*, *B*), we denote by *d_DCJ_*(*C*) the minimum number of DCJ operations required to do a separate DCJ-sorting in *C*, applying DCJs on vertices of *C* (or vertices that result from DCJs applied on vertices that were in C). It is possible to do a separate DCJ-sorting using only optimal DCJs in any component of *AG* (*A*, *B*), thus, in other words, *d_DCJ_*(*A*, *B*) = *∑_C_*_∈_*_AG_*_(_*_A_*_,_*_B_*_)_*d_DCJ_*(*C*) [2]. In [6] we have already defined the *indel-potential* of a component, denoted by λ(*C*), that is the minimum number of runs that we can obtain by DCJ-sorting *C* with optimal DCJ operations only, and can be computed with the formula given in the next proposition.

**Proposition 3** ( [6]**)***Given a component C in AG*(*A*, *B*), *we have*, *if* Λ(*C*) ≥ 1*. Otherwise λ*(*C*) = 0.

Similarly, here we denote by *σ*(*C*) the *substitution-potential* of a component *C*, that is the minimum number of substitutions that we can obtain by DCJ-sorting *C* with optimal DCJ operations only. In order to find a formula to compute *σ*(*C*), we first obtain a stronger version of Proposition 1 where not only the labels of a run are accumulated into a single -adjacency, but pairs of consecutive runs are accumulated into adjacent -adjacencies (that are -adjacencies connected by a single edge in the adjacency graph).

**Proposition 4** ( [6]**)***If γ*_1_*γ*_2_* is a clean**-adjacency in a DCJ-unsorted component C of AG*(*A*, *B*), *such that neither γ*_1 _*nor γ*_2_* are telomeres*, *then it is always possible to extract a clean cycle from C with an optimal DCJ operation*.

**Proposition 5*** Two consecutive runs in a component C can be entirely accumulated into the labels of two adjacent**-adjacencies of C with optimal DCJs*.

*Proof:* By Proposition 1 we assume that two consecutive runs of C are accumulated into -adjacencies *v_A_* and *v_B_*. If *v_A_* and *v_B_* are not adjacent, there are only clean -adjacencies between *v_A_* and *v_B_* in *C*. By Proposition 4, we can apply optimal DCJs to extract clean cycles until *v_A_* and *v_B_* are adjacent.

Pairs of consecutive runs that are accumulated into adjacent -adjacencies can be extracted into a labeled DCJ-sorted component, that can be sorted with one substitution. Observe that minimizing the number of pairs of consecutive runs is equivalent to minimizing the total number of runs. Hence, we can determine the substitution-potential from the indel-potential.

**Proposition 6*** Given a component C in AG* (*A*, *B*), *we have*,* if* Λ(*C*) ≥ 1. *Otherwise σ*(*C*) = 0.

*Proof:* By Proposition 5 we can assume that the runs of *C* are accumulated into pairs of adjacent -adjacencies. By Proposition 3, we can obtain  runs doing a separate DCJ-sorting in *C* with optimal DCJs. Moreover, these optimal DCJs can be done in such a way that pairs of runs that were accumulated into adjacent -adjacencies remain in these adjacent -adjacencies. Since each one of these pairs can be sorted with one substitution, the substitution-potential of *C* is equal to the number of pairs of labeled adjacent -adjacencies, which is:

The formulas to compute λ(*C*) and *σ*(*C*), given in Propositions 3 and 6 above, are indeed very similar. Consequently, many of the results obtained in [6] can be adapted to the new substitution-potential. Let *σ*_0_ and *σ*_1_ be, respectively, the sums of the number *σ* for the components of the adjacency graph before and after a DCJ operation *ρ*. We then define *∆σ*(*ρ*) = *σ*_1_*– σ*_0_. Furthermore, let *∆_dcj_*(*ρ*) be respectively 0, +1 and +2 depending whether *ρ* is optimal, neutral or counter-optimal. We also define *∆d*(*ρ*) = *∆_dcj_*(*ρ*) + *∆σ*(*ρ*).

**Proposition 7 ***Given a DCJ operation ρ acting on a single component*, *we have ∆d*(*ρ*) ≥ + 2 *if ρ is counter-optimal*, *or ∆d*(*ρ*) ≥ 0 *if ρ is neutral*.

We denote by  the minimum number of DCJs and substitutions required to sort separately a component *C* of *AG* (*A*, *B*). The definition of *σ* and Proposition 7 guarantee that .

Observe that, if *C* is a singleton in the adjacency graph, , corresponding to the insertion or the deletion of the whole chromosome. We do not allow the substitution of a linear by a circular singleton and *vice-versa*. However, each pair composed by a singleton in genome *A* and a singleton in genome *B* (such that both are linear or both are circular) can be sorted with one single substitution, which saves one sorting step per pair. Let *P_L_* and *P_C_* be, respectively, the maximum number of disjoint pairs of linear and circular singletons in the adjacency graph. Together with the DCJ-substitution distance per component, these numbers give a good upper bound for :

**Lemma 1*** Given two genomes A and B without duplicated markers*, *we have:*

The formula given by Lemma 1 above corresponds to the exact distance for a particular set of genomes. Given a -adjacency γℓ○ of a genome *A* such that γ≠○, then *γ* is said to be a *tail* of a linear chromosome in *A*. Two genomes are *co-tailed* if their sets of tails are equal (this includes two genomes composed only of circular chromosomes).

**Theorem 2*** Given two co-tailed genomes A and B without duplicated markers*, *we have:*

However, for non co-tailed genomes the use of DCJs applied to two components of the adjacency graph can lead to a shorter sequence of operations sorting one genome into another, as we will see in the next section.

### The DCJ-substitution distance

Recall that *∆σ*(*ρ*) = *σ*_1_*– σ*_0_, where *σ*_0_ and *σ*_1_ are the sums of the number *σ* for the components of the adjacency graph before and after *ρ*. A DCJ operation *ρ* that acts on two components of the adjacency graph is called *recombination*.

**Proposition 8 ***Given any recombination ρ*, *we have ∆σ*(*ρ*) ≥ –2.

*Proof:* Only the recombinations that decrease or do not change the number of runs (∆Λ ≤ 0) have to be analyzed (we can not have ∆*σ* ≤ –1 if the number of runs increases). Consider the recombination of two paths with *i* and *j* runs, that result in two new paths with *i′* and *j′* runs. The best we can have is when *i* and *j* are multiples of 4, *i′* and *j′* are multiples of 4 minus 1 and ∆Λ = –2, that gives: . The analysis of recombinations involving cycles is analogous.

All recombinations involving at least one cycle are counter-optimal and any counter-optimal recombination has *∆d* ≥ 0, thus only path recombinations can have *∆d* ≤ –1. The following definitions are similar to those given in [6], except that here we have a larger number of labeled path types.

Consider an integer *i* ≥ 0. For a second integer *k* ∈ {1, 3}, let  (respectively ) be a sequence with odd 4*i* + *k* runs, starting and ending with an -run (respectively -run). Similarly for *k* ∈ {2, 4}, let  (respectively ), be a sequence with even 4*i* + *k* runs, starting with an -run (respectively -run) and ending with a -run (respectively -run). An empty sequence (with no run) is represented by *ε*. Then each one of the notations *AA_ε_*, , , , , , , *BB_ε_*, , , , , , , *AB_ε_*, , , , , , ,  and  represents a particular type of path (*AA*, *BB* or *AB*) with a particular structure of runs (*ε*, , , , , , , , or ).

The components on which the cuts are applied are called *sources* and the components obtained after the joinings are called *resultants* of the recombination. The complete set of recombinations with *∆d* ≤ –1 is given in Table 1. In Table 2 we also list recombinations with *∆d* = 0 that create at least one source of recombinations of Table 1. We denote by • an *AB*-path that can not be a source in Tables 1 and 2, such as *AB_ε_*, , , , ,  and .

**Table 1:** Path recombinations that have ∆*d* ≤ –1 and allow the best reuse of the resultants.

sources	resultants	∆*σ*	∆_*dcj*_	∆*d*
	• + •	–2	0	–2

		–2	+1	–1
		–2	+1	–1

		–2	+1	–1
		–2	+1	–1
		–2	+1	–1
		–2	+1	–1

		–1	0	–1
		–1	0	–1
		–1	0	–1
		–1	0	–1

	• + •	–1	0	–1
	• + •	–1	0	–1

	• + •	–1	0	–1

	• + •	–1	0	–1
	• + •	–1	0	–1
	• + •	–1	0	–1
	• + •	–1	0	–1

	• + •	–1	0	–1
	• + •	–1	0	–1

	• + •	–1	0	–1
	• + •	–1	0	–1
	• + •	–1	0	–1
	• + •	–1	0	–1

	• + •	–1	0	–1
	• + •	–1	0	–1
	• + •	–1	0	–1
	• + •	–1	0	–1

	• + •	–2	+1	–1

**Table 2:** Recombinations that have ∆*d* = 0 and create resultants that can be used in recombinations with ∆*d* ≤ –1 (listed in Table 1).

sources	resultants	**∆** ***σ***	**∆** ***_dcj_***	**∆** ***d***
		–2	+2	0

		–1	+1	0
		–1	+1	0
		–1	+1	0
		–1	+1	0

		–2	+2	0

		–1	+1	0
		–1	+1	0
		–1	+1	0
		–1	+1	0

**Proposition 9 ***The recombinations with ∆d* = 0 *involving cycles or circular singletons cannot create new components that can be used as sources of recombinations listed in Tables 1 and 2*.

The two sources of a recombination can also be called *partners*. Looking at Table 1 we observe that some types of paths have more partners than other types of paths. For example, all partners of  and  paths are also partners of  and  paths. Furthermore, some resultants of recombinations in Tables 1 and 2 can be used in other recombinations. These observations allow the identification of groups of recombinations, as listed in Table 3.

**Table 3:** All recombination groups obtained from Tables 1 and 2 (the recombinations from Table 2 appear only in groups in *Y* and *Z*). The column **scr** indicates the contribution of each path in the distance decrease.

	sources	resultants	**∆** ***d***	scr
*U*		2•	–2	–1

*V*		4•	–3	–3/4
		4•	–3	–3/4

*W*		3•	–2	–2/3
		3•	–2	–2/3
		3•	–2	–2/3
		3•	–2	–2/3
			–2	–2/3
			–2	–2/3
			–2	–2/3
			–2	–2/3

*X*	Recombinations from Table 1 with *∆d* = –1		–1	–1/2

*Y*		4•	–2	–1/2
		4•	–2	–1/2

*Z*		3•	–1	–1/3
		3•	–1	–1/3
		3•	–1	–1/3
		3•	–1	–1/3
		3•	–1	–1/3
		3•	–1	–1/3
		3•	–1	–1/3
		3•	–1	–1/3
			–1	–1/3
			–1	–1/3
			–1	–1/3
			–1	–1/3
			–1	–1/3
			–1	–1/3

The deductions shown in Table 3 can be computed with an approach that greedily maximizes the number of recombinations in *U*, *V*, *W*, *X*, *Y* and *Z* in this order. The *U* part contains only one operation and the two groups in V are mutually exclusive after applying U. The part *W* is then the application of all possible remaining groups of two operations with ∆*d* = –2. Similarly, the part X is only the application of all possible remaining operations with ∆*d* = –1. After X, the two groups in *Y* are mutually exclusive and then the same happens to the groups in Z. Although some groups in *W*, *X* and *Z* have some reusable resultants, those are actually never reused (if operations that are lower in the table use as sources resultants from higher operations, the sources of all referred operations would be previously consumed in operations that occupy even higher positions in the table). Due to this fact, the number of operations in *U*, *V* , *W*, *X*, *Y* and *Z* depends only on the initial number of each type of component.

With the results presented in this section we have an exact formula to compute the DCJ-substitution distance:

Theorem 3 *Given two genomes Aand Bwithout duplicated markers*, *we have*:

*where P_L _and P_C_ are the numbers of disjoint pairs of linear and circular singletons and U*, *V*, *W*, *X*, *Y and Z are computed as described above*.

The formula given in Theorem 3 is analogous to the one which we have obtained in [6] to compute the DCJ-indel distance. Both formulas depend on factors that can be computed in linear time [6].

#### Triangular inequality

Note that, since only unique markers can be substituted in this model, we avoid the “free lunch problem”, mentioned in [5], that is the possibility of transforming any genome A into any genome *B* by simply substituting the whole content of A by the whole content of B. However, the triangular inequality can be disrupted in the DCJ-substitution distance. In other words, given any three genomes *A*, *B* and *C* without duplicated markers, there is no guarantee that the triangular inequality  holds. In a companion paper [11] we provide an efficient way to establish the triangular inequality *a posteriori* in both the DCJ-indel [6] and the DCJ-substitution distances.

### Experiments

The objective of this model is to provide a parsimonious genomic distance, that in practice is a lower bound to real distances. Nevertheless, we could run some experiments on data from human *X* and *Y* chromosomes and obtained a parsimonious sorting scenario that is coherent with the results available in the literature. During evolution, a portion of the human *Y* chromosome has become increasingly subjected to local mutations, while the X chromosome remained relatively conserved, as we will see in the following. Human X and Y chromosomes are very different and, while X is 155 Mbp long, the Y chromosome is 58 Mbp long. However, they still share *pseudo-autosomal* regions at both extremities and are believed to have evolved from an identical autosomal pair [12] (the autosomes are all non-sex chromosomes). Current theories suggest that the pseudo-autosomal region, which originally covered the whole chromosomes, was successively pruned by a few big inversions on the Y chromosome [13] (we call these inversions *pruning*). After each pruning inversion, several mutations seem to have occurred on the affected part of the Y chromosome, while X remained “closer” to the common ancestor.

**Figure 5:**
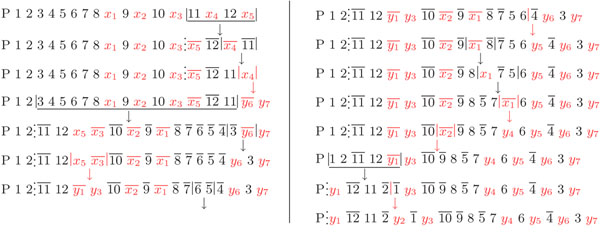
A parsimonious scenario of 8 inversions and 6 substitutions (including 2 insertions and 1 deletion) sorting human X into Y chromosome, using the dataset given in [10]. The symbol ‘P’ represents the current pseudo-autosomal region in the beginning of X and Y. Each number represents a common marker, each symbol *x_i_* represents a unique marker in X and each symbol *y_i_* represents a unique marker in Y (the unique markers were also obtained from the data in [10]). The three pruning inversions suggested in [[10], Fig. 7] are underlined. The boundary of the pseudo-autosomal region, indicated with vertical dots, is shifted to the left after each pruning inversion.

A parsimonious scenario of 8 inversions on the markers common to chromosomes X and Y has been published in [[10], Fig. 7], and is given as an argument to support the existence and bounds of the three most recent pruning inversions, but unique markers were simply ignored. We used our method to compute the DCJ-substitution distance using the same dataset, but reincorporating the unique markers, and obtained a DCJ-substitution distance of 14. Then we manually reconstructed the evolutionary scenario of human chromosomes X and Y and obtained a parsimonious scenario with 8 inversions and 6 substitutions (including 2 insertions and 1 deletion) that is coherent with the pruning inversions given in [10] (see Figure 5). Although a DCJ is a very comprehensive operation and can represent many rearrangement events, in the analysis of unichromosomal genomes DCJs often represent only inversions, and this also happens in this dataset.

### Discussion

Our method was designed to find gene mutations, but it could also help to improve orthology assignments, that are the computational prediction of orthologous pairs of genes from different species. No orthology predictor is able to find all assignments correctly. In particular, when comparing two different species, some pairs of orthologous genes that are below the predictor threshold remain unassigned. Since our substitutions establish a relation between different genes in the two compared genomes, they correspond to candidates to be assigned as orthologous genes.

## Conclusions and future work

In this work we presented a new model to compare two genomes with unequal content, but without duplicated markers, using substitutions and DCJ operations, and developed a linear time algorithm to exactly compute the DCJ-substitution distance.

Although the objective of this model is to provide a parsimonious genomic distance, that in practice is a lower bound to real distances, based on our method we have manually reconstructed a parsimonious evolutionary scenario of human chromosomes X and Y. We considered biological constraints that are specific to this case and obtained a scenario that is coherent with the results given in the literature.

By reconstructing a parsimonious scenario that minimizes substitutions, we may identify genomic regions that were subject to continuous mutations during evolution and could have a common evolutionary origin. Currently our method is only able to compute the genomic distance, but in a future work we intend to study the space of all parsimonious sorting scenarios and develop methods to systematically identify such regions.

The DCJ-substitution model could also be used to refine orthology assignments, since in some cases a substitution could actually correspond to an unannotated orthology. We also plan on exploring the use of our method in refining orthology in a future work.

## Competing interests

The authors declare that they have no competing interests.

## Authors’ contributions

MDVB and JS have elaborated the model. MDVB, RM, LCR and JS have proved the results and written the paper. MDVB has also run the experiments.
